# Efficacy of the monoclonal antibody EGFR inhibitors for the treatment of metastatic colorectal cancer

**DOI:** 10.3747/co.v17is1.616

**Published:** 2010-07

**Authors:** M. Fakih, R. Wong

**Affiliations:** * Department of Medicine, Roswell Park Cancer Institute, Buffalo, New York; † Cancer Care Manitoba, Winnipeg, Manitoba

**Keywords:** BRAF, KRAS, EGFR, colorectal carcinoma, cetuximab, panitumumab

## Abstract

Two anti-epidermal growth factor receptor (EGFR) monoclonal antibodies (MoAbs) have been approved in Canada for the treatment of metastatic colorectal cancer (mCRC) – cetuximab, a mouse-human chimeric MoAb, and panitumumab, a fully human MoAb. This paper reviews the efficacy of the anti-EGFR monoclonal antibodies cetuximab and panitumumab – both as monotherapy and in combination with cytotoxic chemotherapy – in the treatment of mCRC. Both cetuximab and panitumumab have demonstrated clinical efficacy in monotherapy in patients with mCRC, an advantage that has recently been found to be limited largely to those with wild-type KRAS tumors. Advantages of using these agents in monotherapy include reduced cost and toxicity. While the addition of cetuximab to irinotecan has shown superior progression-free survival and response compared with cetuximab monotherapy, there is currently no evidence for a benefit of panitumumab in combination with irinotecan.

## 1. INTRODUCTION

There are an estimated 22,000 new cases of colorectal cancer (CRC) each year in Canada. CRC is the second leading cause of death from cancer after lung cancer, accounting for more than 9,000 deaths each year^1^. When diagnosed in the early stages of disease, CRC is associated with a five-year survival rate of up to 90%^2^. However, for those with metastatic CRC (mCRC), which represents approximately 20% of first diagnoses, the five-year survival rate is only 10%^2^,^3^. With the introduction of treatments involving irinotecan or oxaliplatin in combination with fluorouracil and leucovorin, survival has improved for patients with mCRC over the past decade^4^–^17^. However, as most patients eventually develop resistance to these therapies, new active treatment options in this setting were needed.

Insights into the molecular pathogenesis of CRC prompted the development of specific target-directed therapies for the treatment of mCRC, including monoclonal antibodies (MoAbs) that target the epidermal growth factor receptor (EGFR). The two anti-EGFR MoAbs approved in Canada for the treatment of mCRC are cetuximab, a mouse-human chimeric MoAb, and panitumumab, a fully human MoAb.

## 2. RATIONALE FOR THE USE OF ANTI-EGFR MOABS IN THE TREATMENT OF MCRC

EGFR – also known as HER-1 or erb-B1 – is a ubiquitous transmembrane glycoprotein that contains an amino-terminal extracellular ligand-binding domain, a hydrophobic transmembranous region, and a cytoplasmic domain. Within the cytoplasmic domain there is a tyrosine kinase domain and a carboxy-terminal region, which contains critical tyrosine residues and receptor regulatory motifs^18^.

EGFR is abnormally activated in a number of human malignancies, through several mechanisms, including receptor overexpression, gene amplification, activating mutations, overexpression of receptor ligands, and/or loss of negative regulatory mechanisms^19^. EGFR activation of the receptor leads to recruitment and phosphorylation of several intracellular substrates which, in turn, engage tumorpromoting activities^19^.

Elevated EGFR expression has been documented in 60% to 80% of patients with mCRC^20^,^21^, and correlates with disease progression, metastatic spread, and poorer prognosis^22^. Pharmacologic interventions have therefore been developed to target the molecular and cellular consequences of EGFR alterations^23^,^24^. One such intervention has been the use of MoAbs that compete with the binding of activating ligands to the extracellular domain of the receptor.

Two MoAbs that bind and block EGFR signaling are currently approved in Canada for the treatment of mCRC. The first drug approved in this class was cetuximab (Erbitux™; Bristol-Myers Squibb/Im-Clone Systems Incorporated, Montreal, Quebec), an immunoglobulin (Ig) G1 human-mouse chimeric MoAb. Cetuximab is approved both as a monotherapy and in combination with irinotecan for the treatment of mCRC that is refractory to other irinotecan-based chemotherapy regimens^25^. Panitumumab (Vectibix™, Amgen, Mississauga, Ontario) is an IgG2 fully human MoAb directed against the EGFR, and has been approved for use as monotherapy for the treatment of EGFR-expressing mCRC with wild-type *KRAS* after failure of fluoropyrimidine-, oxaliplatin-, and irinotecan-containing chemotherapy regimens^26^.

Cetuximab and panitumumab bind to the easily accessible extracellular domain of the receptor and compete with ligand binding. This blocks the downstream signaling of EGFR, resulting in impaired cell growth and proliferation^25^–^28^. In addition, cetuximab has been suggested to induce antibody-mediated cellular cytotoxicity (ADCC) due to its human IgG1 backbone, which may contribute to its anti-tumor effects^29^,^30^.

## 3. EFFICACY OF THE ANTI-EGRF MOABS

### 3.1 Cetuximab

#### 3.1.1 Cetuximab monotherapy

A series of phase II and phase III trials have evaluated the efficacy of cetuximab monotherapy in the treatment of patients with mCRC who failed to respond to previous treatment with irinotecan^31^–^34^. In the first phase II open-label trial^31^, 57 patients with EGFR-expressing mCRC who had been unresponsive to previous treatment with irinotecan were given cetuximab by weekly intravenous infusion at the standard dosage – 400 mg/m^2^ over 2 hours in the first dose, followed by subsequent weekly treatments of 250 mg/m^2^ over 1 hour. Sixteen patients (28%) had received one prior regimen for their disease before study entry. Forty-one patients (72%) had received two or more chemotherapy regimens (including adjuvant regimens, if given) for their disease before study entry. A partial response was observed in six patients (10.5%; 95% CI 4–22%). Twenty additional patients experienced a minor response, defined as a tumor reduction of 25% to 49%, or stable disease, defined as either growth or shrinkage of less than 25% lasting for a minimum of 12 weeks from the start of treatment. The median time to tumor progression was 1.4 months, with a median survival of 6.4 months from the initiation of cetuximab.

Among 346 patients with EGFR-expressing mCRC refractory to irinotecan, oxaliplatin, and fluoropyrimidines, cetuximab given at the standard dosage elicited a response rate of 12.4% (95% CI 9.1–16.4%)^32^. Median progression-free survival (PFS) and overall survival (OS) times were 1.4 months (95% CI 1.4–2.1 months) and 6.6 months (95% CI 5.6–7.6 months), respectively. Patients enrolled in this study had received a median of four prior chemotherapy regimens (range 2 to 9). All patients had been treated with both irinotecan- and oxaliplatin-based regimens, with 93.6% and 98.3% developing progressive disease during treatment or within three months of treatment with these agents, respectively, in the metastatic setting.

In comparison with best supportive care (BSC), cetuximab has demonstrated significant improvements in survival in patients with mCRC. In the phase III CO.17 trial conducted by the National Cancer Institute of Canada Clinical Trials Group (NCIC CTG) and the Australasian Gastro-Intestinal Trials Group (AGITG), Jonker et al. randomly assigned 572 patients with EGFR-expressing mCRC refractory to fluoropyrimidine, irinotecan, and oxaliplatin to standard-dose cetuximab plus BSC (n = 287) or BSC alone (n = 285), which was defined as “measures designed to provide palliation of symptoms and improve quality of life as much as possible”^33^. Approximately 37% of patients enrolled in the study had received adjuvant therapy. The number of previous regimens, including adjuvant, was roughly 17% for one to two lines, 38% for three lines and 45% for four or more lines of therapy. Compared with BSC alone, cetuximab significantly improved OS (hazard ratio [HR] 0.77; 95% CI 0.64–0.92; *p* = 0.005) and PFS (HR 0.68; 95% CI 0.57–0.80; *p* < 0.001). Median OS was 6.1 months in the group treated with cetuximab compared with 4.6 months in those receiving BSC alone. Twenty-three patients (8.0%) in the cetuximab group had partial responses compared with none in those assigned to BSC alone (*p* < 0.001); an additional 31.4% of patients treated with cetuximab and 10.9% of patients assigned to BSC alone had stable disease (*p* < 0.001).

Cetuximab was also shown to preserve quality of life to a greater extent than BSC. Compared with those who received BSC alone, patients treated with cetuximab experienced less deterioration in physical function at eight weeks (mean change score, −3.9 vs. −8.6; *p* < 0.05) and 16 weeks (mean change score, −5.9 vs. −12.5; *p* = 0.03), and less deterioration in global health status at eight weeks (mean change score, −0.5 vs. −7.1; *p* = 0.008) and 16 weeks (mean change score, −3.6 vs. −15.2; *p* < 0.001)^33^.

Because cetuximab targets the EGFR, most clinical trials of cetuximab in the treatment of mCRC include patients with EGFR-positive tumors, as determined by immunohistochemistry. However, the intensity of EGFR immunostaining has not been shown to be related to the activity of cetuximab, and objective responses have been reported in patients with EGFRnegative tumors^35^. An ongoing phase II, multicentre study is evaluating the efficacy of cetuximab in the absence of detectable EGFR expression^34^. Patients with refractory, EGFR-undetectable mCRC who had undergone at least one standard chemotherapy regimen containing fluoropyrimidine were given cetuximab in the standard dosage regimen and evaluated for tumor response every six weeks. Preliminary results presented at the *2008 Annual Meeting of the American Society of Clinical Oncology* (ASCO) revealed partial responses in six of the 85 patients who were evaluable for response (ORR 7%; 95% CI 3%–15%)^34^. These preliminary results suggest that cetuximab monotherapy is comparably active in both EGFR-detectable and EGFR-undetectable disease, confirming the unreliability of EGFR immunohistochemistry as an indicator of cetuximab activity.

#### 3.1.2. Cetuximab combination therapies

Cetuximab has also been evaluated in the first- and second-line treatment of mCRC in combination with cytotoxic chemotherapies.

A phase I/II study evaluated the safety and efficacy of cetuximab in combination with FOLFIRI (irinotecan, leucovorin, fluorouracil) in 52 patients with previously untreated, unresectable mCRC^36^. The combination of cetuximab and FOLFIRI was active and well tolerated, with an overall response rate of 48%, a median PFS of 8.6 months, and an OS of 22.4 months. For those with initially unresectable metastases, treatment with cetuximab and FOLFIRI allowed 27% to undergo resection, resulting in the elimination of residual tumor in 71% of these patients. Because of these resections, an accurate estimation of PFS was not possible, but these promising results prompted the design of the phase III CRYSTAL (Cetuximab Combined With Irinotecan in First-Line Therapy for Metastatic Colorectal Cancer) trial, which examined the efficacy of FOLFIRI in combination with cetuximab for the first-line treatment of mCRC^37^. The CRYSTAL trial randomly assigned 1198 patients with EGFR-positive mCRC to receive cetuximab plus FOLFIRI (n = 599) or FOLFIRI alone (n = 599). Following an updated analysis of the data in the ITT population, OS was found to be significantly longer in patients receiving cetuximab plus FOLFIRI compared with FOLFIRI alone (median OS, 19.9 months vs. 18.6 months, HR 0.878, 95% CI 0.774–0.995, *p* = 0.042). Among patients with wild-type *KRAS* tumors, OS (HR=0.796, 95% CI 0.670–0.946, *p* = 0.0094) and PFS (HR=0.696, 95% CI 0.558–0.867, *p* = 0.0012) were significantly greater with the addition of cetuximab to FOLFIRI than with FOLFIRI alone^38^.

The OPUS trial compared the response rates of FOLFOX-4 (leucovorin, fluorouracil, oxaliplatin) plus cetuximab with those of FOLFOX-4 alone for the first-line treatment of EGFR-positive mCRC^39^. Patients were randomly assigned in a 1:1 ratio to treatment with standard-dosage cetuximab plus FOLFOX-4 or to FOLFOX-4 only. Among patients with wild-type *KRAS* tumors, PFS was significantly longer (8.3 months vs. 7.2 months, HR 0.567; 95% CI 0.375–0.856; *p* = 0.0064) and OR was significantly higher (57% vs. 34%, odds ratio 2.5512, 95% CI 1.3799–4.7169; *p* = 0.0027) with the combination of cetuximab plus FOLFOX4 than with FOLFOX4 alone^40^. The addition of cetuximab to FOLFOX4 was associated with a four-month improvement in OS (18.5 to 22.8 months) on KRAS wild-type patients; this difference was not statistically significant, likely because of the small number of patients available for analysis. However, in a recent meta-analysis of the OPUS and CRYSTAL trials, OS was significantly longer in patients with wild-type *KRAS* tumors treated with cetuximab plus chemotherapy than in those receiving chemotherapy alone (HR 0.81; 95% CI 0.69–0.94; *p* = 0.006)^41^. The addition of cetuximab to chemotherapy was also found to reduce the risk of disease progression by 34% (HR 0.66; 95% CI 0.55–0.80; *p* < 0.0001) and increase the likelihood of response by more than twofold (OR 2.16; 95% CI 1.64–2.86; *p* < 0.0001) compared with chemotherapy alone in patients with wild-type *KRAS* tumors.

The EPIC (Erbitux Plus Irinotecan for Metastatic Colorectal Cancer) study investigated whether adding cetuximab to irinotecan following failure of prior treatment with fluoropyrimidine and oxaliplatin would prolong survival in irinotecan-naive patients with EGFR-expressing mCRC^42^. This open-label, phase III study randomly assigned 1298 patients with EGFR-expressing mCRC who had experienced first-line treatment failure with fluoropyrimidine and oxaliplatin to treatment with cetuximab plus irinotecan or irinotecan alone. The combination of cetuximab and irinotecan was associated with a 31% reduction in the risk of progression compared with irinotecan alone (*p* ≤ 0.0001, HR 0.692, 95% CI 0.617–0.776) and resulted in significantly better scores in the QOL analysis of global health status (*p* = 0.047). The response rate was also greater with cetuximab and irinotecan (16.4%, 95% CI 13.6–19.4) than with irinotecan alone (4.2%, 95% CI 2.8–6.0) (*p* < 0.0001). Complete responses were observed in nine patients receiving the combination versus one patient receiving irinotecan alone. The primary endpoint of OS was comparable between the two treatment groups, which was potentially confounded by the fact that 46.9% of patients in the irinotecan arm went on to receive cetuximab.

**Figure 1:**
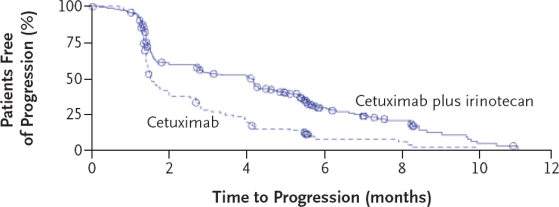
Time to disease progression in patients treated with cetuximab alone or cetuximab plus irinotecan in the BOND trial^20^

**Figure 2:**
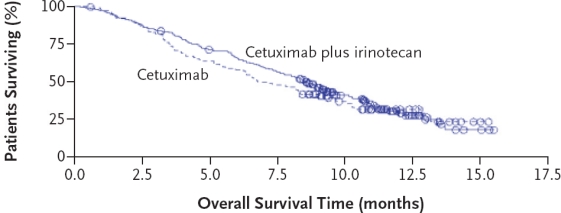
Overall survival in patients treated with cetuximab alone or cetuximab plus irinotecan in the BOND trial^20^

The open-label, randomized BOND trial^20^ compared the efficacy of cetuximab in combination with irinotecan with that of cetuximab alone in mCRC that was refractory to treatment with irinotecan. The BOND trial enrolled 329 patients with EGFR-expressing mCRC whose disease had progressed despite treatment with an irinotecan-based regimen. Patients from 56 centers in 11 European countries were randomly assigned to receive either cetuximab and irinotecan (n = 218) or cetuximab monotherapy (n = 111 patients). In the event of disease progression, patients on cetuximab monotherapy were permitted to receive additional treatment with irinotecan. In this study, nearly 80% of the patients who underwent randomization had received two or more previous regimens. The overall response rates were 22.9% (95% CI 17.5–29.1%) in the combination therapy group and 10.8% (95% CI, 5.7–18.1%) in the monotherapy group (*p* = 0.007), with median durations of response of 5.7 months and 4.2 months, respectively. Disease control (complete response plus partial response plus stable disease) was achieved in 55.5% and 32.4% of patients receiving combination treatment and cetuximab monotherapy, respectively (*p* < 0.001). Among those who had progressed during or within one month after irinotecan therapy, the response rates were 25.2% (95% CI, 18.1–33.4%) and 14.1% (95% CI, 7.0 −24.4%) in the combination therapy and monotherapy groups, respectively (*p* = 0.07). Of note, treatment with cetuximab was found to be as effective in patients who had previously received oxaliplatin in addition to irinotecan before entering the study, with response rates of 22.2% and 8.5% in the combination therapy and monotherapy groups, respectively (*p* = 0.01). In the intention-to-treat analysis, the median time to progression of disease was 4.1 months in the combination-therapy group and 1.5 months in the monotherapy group (HR 0.54, 95% CI 0.42–0.71), indicating a 46% reduction in the risk of progression with combination therapy compared with monotherapy (*p* < 0.001) (Fig. 1). OS was not significantly different between the two groups (Fig. 2).

The effectiveness of irinotecan in combination with cetuximab in patients with irinotecan-refractory mCRC suggests that cetuximab may have the ability to circumvent irinotecan resistance. It has been postulated that cells acquire irinotecan resistance through several mechanisms^43^, and cetuximab may overcome this resistance by inhibiting EGFR, thereby preventing drug efflux^43^–^48^, restoring apoptosis^49^ or impairing DNA-repair activity^50^,^51^.

The COIN trial evaluated whether the addition of cetuximab to continuous oxaliplatin-based chemotherapy improves OS in the first-line treatment of mCRC. The 1630 patients in the study received one of two oxaliplatin-based regimens chosen by their physicians – either 5-fluorouracil (5FU) or capecitabine. An unexpected result of COIN was the lack of benefit in OS or PFS with the addition of cetuximab in patients with wild-type *KRAS* tumors; however, there was a significant increase in best overall response (*p* = 0.04). The chemotherapy used by over two-thirds of patients in COIN was XELOX, which includes two cytotoxic agents – capecitabine (Xeloda) and oxaliplatin. The other chemotherapy used in COIN was FOLFOX, consisting of bolus and infusional 5FU, folinic acid and oxaliplatin. While analyses of the capecitabine arm are not yet complete, results have shown higher rates of non-hematological toxicity leading to greater dose reductions in 47% of patients – from 1000 mg/m^2^ to 850 mg/m^2^, a potentially suboptimal therapeutic dose in mCRC. As well, 9% of patients in COIN older than 75 years, and 8% had ECOG 2 performance status. A trend toward improvement in PFS for the cetuximab arm was seen only in the FOLFOX group. This may suggest that the compounded toxicities seen in combination with XELOX overcame the potential benefits in terms of prolongation of disease control (dose reductions and delay). Further analyses are needed to examine issues of efficacy and toxicity related to the combination of cetuximab and capecitabine, and to further understand the impact of population characteristics on the overall results^52^.

Cetuximab has also been investigated in combination with the MoAb bevacizumab in the first-line treatment of mCRC, with conflicting results. The BOND2 study showed that the combination of bevacizumab and cetuximab, with or without irinotecan, improved response rates and time to tumor progression in heavily pretreated, irinotecan-refractory mCRC^53^. The CAIRO2 trial randomly assigned 755 patients with previously untreated mCRC to capecitabine, oxaliplatin, and bevacizumab (n = 378) or to the same regimen plus the addition of weekly cetuximab (n = 377)^54^. The addition of cetuximab to capecitabine, oxaliplatin, and bevacizumab resulted in significantly shorter PFS (9.4 months vs. 10.7 months, *p* = 0.01) and inferior quality of life scores.

## 4. PANITUMUMAB

### 4.1 Panitumumab monotherapy

Panitumumab has demonstrated clinical activity as a single agent in patients with mCRC who have progressed on chemotherapy. The anti-tumor activity of panitumumab was established in a multicentre, phase II study of mCRC patients with EGFR tumor expression levels of 10% or higher who had progressed during or following treatment with fluoropyrimidine, irinotecan, and oxaliplatin treatment^15^. Patients were treated with panitumumab 6 mg/kg every two weeks until disease progression. Of the 39 patients included in the efficacy data set, three (8%) had a partial response, eight (21%) had stable disease, and 19 (49%) had disease progression at 16 weeks.

In a phase II open label, multicentre study, 148 patients with EGFR-positive mCRC underwent weekly treatment with panitumumab 2.5 mg/kg, resulting in a response rate of 9% and PFS of 14 weeks^55^. The response rate was not affected by the number of prior treatment regimens nor by the level of EGFR staining. These results are supported by data from a phase II multicentre, single-arm study showing a response to panitumumab in patients with low or negative EGFR tumor cell expression^56^. Patients with chemorefractory mCRC and low (< 1%) or negative (1% to 9%) EGFR tumor cell expression were treated with panitumumab 6 mg/kg every two weeks until disease progression or intolerability. Overall responses were similar between the low-EGFR and negative-EGFR groups, as were median time to response, duration of response, and incidence of adverse events^56^.

The effect of panitumumab monotherapy has also been compared with BSC in the treatment of EGFR-positive mCRC^57^. Patients were randomly assigned to panitumumab 6 mg/kg given every two weeks along with BSC (n = 231) or BSC alone (n = 232). Approximately 37% of patients had received prior adjuvant therapy. One hundred percent and approximately 37% of patients had received two and three prior lines of therapy, respectively. Six percent of patients in each arm received more than three prior lines of therapy; these patients were enrolled before a protocol amendment that limited entry criteria to two to three prior lines of therapy. PFS was significantly improved by panitumumab, with a mean PFS of 13.8 (SE 0.8) weeks, compared with 8.5 (SE 0.5) weeks for BSC (HR 0.54; 95% CI 0.44–0.66, *p* < 0.0001). Objective response rates were also improved by panitumumab, with response rates of 10% for panitumumab and 0% for BSC after a 12-month minimum follow-up (*p* < 0.0001). An additional 62 (27%) patients in the panitumumab group and 23 (10%) patients in the BSC group had a best response of stable disease, with similar results seen in the group of patients who were permitted to cross over to panitumumab following progression on BSC alone. In an exploratory analysis, in which patients with stable disease were removed from the panitumumab group, approximately 80% of the overall treatment effect on PFS was attributed to nonresponders, suggesting that stable disease is associated with a significant clinical benefit in this patient population. An OS benefit was not observed, likely confounded by the similar activity of panitumumab after 76% of patients in the BSC group entered into the crossover study. In the extension phase of the study^58^, 176 patients whose disease had progressed while receiving BSC in the phase III study received one or more dose of panitumumab. Objective responses were observed in 11% of patients, and an additional 33% of patients had a best response of stable disease, with a disease control rate of 44%.

### 4.2 Panitumumab combination therapies

Studies evaluating panitumumab for the first- and second-line treatment of mCRC in combination with cytotoxic chemotherapies are limited. The phase II multicentre, single-arm PACCE (Panitumumab Advanced Colorectal Cancer Evaluation) study evaluated the efficacy and safety of adding panitumumab to combination chemotherapy with bevacizumab for the first-line treatment of mCRC^59^. The PACCE trial was stopped early when a planned interim analysis revealed that both PFS and OS were better in the standard chemotherapy arm versus the panitumumab plus chemotherapy arm. In contrast to the CAIRO2 trial, which evaluated the combination of cetuximab and bevacizumab in the treatment of mCRC, in the PACCE trial, worse outcomes were seen in the panitumumab group.

The recently completed PRIME study was the first global, phase III trial to investigate the combination of an anti-EGFR monoclonal antibody with FOLFOX as first-line treatment for patients with mCRC^60^. In PRIME, 1183 patients were randomly assigned in 1:1 ratio to biweekly treatment with either panitumumab 6.0 mg/kg plus FOLFOX4 or FOLFOX4 alone. When administered in combination with FOLFOX, panitumumab significantly prolonged PFS compared with FOLFOX alone (9.6 months vs. 8.0 months; HR = 0.80; 95% CI 0.66–0.97; *p* = 0.0234) in the first-line treatment of patients with *KRAS* wild-type mCRC. Median OS was prolonged by 4.2 months with the addition of panitumumab to FOLFOX compared with FOLFOX alone (23.9 months vs. 19.7). However, this difference did not reach statistical significance (HR=0.83, *p* = 0.072) (Amgen, unpublished data).

The phase III 181 trial evaluated the efficacy of adding panitumumab to FOLFIRI as second-line therapy for mCRC^61^. A total of 1186 patients were randomly assigned to treatment with biweekly panitumumab 6.0 mg/kg plus FOLFIRI (Arm 1) (n = 591) or FOLFIRI alone (Arm 2) (n = 595). For patients with wild-type *KRAS*, the combination of panitumumab and FOLFIRI improved median PFS (5.9 months vs. 3.9 months; HR 0.73; 95% CI 0.593–0.903; *p* = 0.004) and response rate (by blinded central review) (35% vs. 10%) compared with FOLFIRI alone. Median OS was not significantly different among the two groups, and there was no difference in PFS, OS, or response rate among patients with mutant *KRAS*.

A randomized, phase III study has also evaluated the efficacy of adding panitumumab to FOLFIRI in the second-line treatment of mCRC^62^. A total of 1186 patients with metastatic adenocarcinoma of the colon or rectum, documented disease progression of six months or less after one prior therapy with fluoropyrimidine for mCRC, and an ECOG score of 0–2 were randomly assigned to treatment with panitumumab plus FOLFIRI or FOLFIRI alone. Among patients with wild-type *KRAS* tumors, PFS was significantly increased from 3.9 months to 5.9 months (HR 0.73; 95% CI 0.59–0.90; *p* = 0.004), and there was a non-significant increase in OS from 12.5 months to 14.5 months (HR 0.85; 95% CI 0.70–1.04; *p* = 0.12) with the addition of panitumumab to FOLFIRI compared with FOLFIRI alone.

## 5. ALTERNATIVE DOSING REGIMENS FOR CETUXIMAB AND PANITUMUMAB

Cetuximab is currently approved in a weekly dosing regimen^25^. However, as many chemotherapy regimens are administered every second week, it would be convenient and cost-effective if cetuximab administration could be coordinated with the chemotherapy schedule^63^. Preliminary results from a two-part, phase I study of patients with EGFR-expressing mCRC who had not received previous chemotherapy demonstrated that the pharmacokinetics of cetuximab were comparable between a weekly 250 mg/m^2^ regimen and a regimen of cetuximab 500 mg/m^2^ given every two weeks^63^. These data prompted a group in Denmark to begin treating a group of patients with nonresectable mCRC who had failed to respond to 5-fluorouracil, irinotecan, and oxaliplatin, according to a biweekly treatment schedule of cetuximab and irinotecan^64^. Among the initial 74 patients treated according to this dosing schedule for one year, 1% and 24% of patients experienced complete and partial responses, respectively, compared with 0% and 19% in a control group receiving the standard weekly dosing regimen. Median PFS was 5.4 months with both the biweekly and weekly regimens, and OS was 8.9 months and 10.4 months in the biweekly and weekly groups, respectively (Table 1). These efficacy data are similar to the efficacy of the weekly regimen described in other studies^20^,^63^,^65^. Toxicity data were also comparable with those from studies where cetuximab is administered on a weekly basis^20^,^66^. Salvage therapy with simplified biweekly cetuximab plus irinotecan may therefore offer a convenient, effective and well-tolerated regimen in patients with mCRC who are resistant to 5-fluorouracil, irinotecan, and oxaliplatin^20^,^66^. An ongoing phase II study is underway to confirm these findings.

Similar results were seen in a phase II, institutional exploratory trial of irinotecan and cetuximab given in a biweekly dosing regimen to patients with mCRC who had disease progression following at least one previous line of chemotherapy^67^. Forty patients were treated with irinotecan 180 mg/m^2^ and cetuximab 500 mg/m^2^ every two weeks until unacceptable toxicity or progressive disease. With two complete responses and seven partial responses, the overall response rate was 22.5%. The disease control rate was 60%, time to progression was 3.4 months, and OS was eight months. Toxicities were favorable compared with weekly cetuximab combination schedules.

These preliminary data suggest that biweekly dosing of cetuximab may offer a convenient strategy for the treatment of mCRC, without compromising efficacy.

Extended dosing strategies have also been explored with panitumumab. Panitumumab is currently approved for biweekly administration, and its safety and efficacy for administration every three weeks have been explored^68^,^69^. A phase I study of 96 patients with various tumor types randomly assigned patients to treatment with one of three dosing schedules of panitumumab – 2.5 mg/kg weekly, 6.0 mg/kg every two weeks, and 9.0 mg/kg every three weeks. The minimal serum panitumumab concentrations were similar among the three dosing strategies, with steady-state reached after six weeks for all schedules. These data suggest that panitumumab may be flexibly dosed from weekly to every three weeks^69^. However, another study showed higher rates of several grade 3 adverse events (erythema, pruritus, acneiform dermatitis, fatigue, hypomagnesemia) with the every three weeks regimen^69^.

## 6. MARKERS OF RESPONSE TO ANTI-EGFR MOAB THERAPY: PATIENT SELECTION

Despite the demonstrated efficacy of the anti-EGFR therapies in the treatment of mCRC, response rates in unselected patient populations have remained low^15^,^20^,^31^–^34^,^36^,^37^,^42^,^55^–^58^, prompting the search for markers to identify patients who are most likely to benefit from anti-EGFR therapy. As described above, EGFR testing by immunohistochemistry has not demonstrated value in predicting which patients will respond to cetuximab or panitumumab^20^,^31^, and objective responses have been observed in patients who do not express EGFR^35^,^55^. Several biologic markers involved in EGFR intracellular signaling pathways have been investigated as potential predictors of response to the anti-EGFR MoAbs.

### 6.1 *KRAS* mutational status

#### 6.1.1 Cetuximab

Recent reports have shown that the presence of mutations on the *KRAS* gene is a strong predictor of nonresponsiveness to cetuximab^70^–^74^. In order to identify gene expressions that correlate with best clinical response to cetuximab, 110 patients with mCRC were enrolled in a monotherapy trial and underwent transcriptional profiling on RNA from mandatory pretreatment metastatic biopsies^71^. Significantly higher rates of disease control were seen in patients whose tumors lacked the *KRAS* mutations than in those with *KRAS* mutations (*p* = 0.0003).

In a follow-up to the phase III CO.17 trial, correlative analysis was used to determine whether the mutation status of the *KRAS* gene modified the effect of cetuximab on OS and PFS in this patient population^73^. Among patients with wild-type *KRAS*, median OS was significantly greater among patients treated with cetuximab (9.5 months) than among those treated with BSC (4.8 months), while patients with *KRAS* mutations did not experience an OS benefit from cetuximab.

Several recent studies have shown that *KRAS* mutational status also plays a key role in response and survival in patients with irinotecan-resistant mCRC who are treated with cetuximab and irinotecan. Among 281 patients from seven series, all 77 responses – including three complete responses and 74 partial responses – were in patients with wild-type *KRAS* tumors^74^.

In the CRYSTAL trial, which showed a reduction in the risk of progression of mCRC with the addition of cetuximab to FOLFIRI, retrospective subgroup analysis revealed that the benefit of cetuximab on tumor response was limited to patients with *KRAS* wild-type tumors^37^. DNA was extracted from 540 available archived tumor samples from the CRYSTAL trial to determine *KRAS* mutation status^75^. Among patients with wild-type *KRAS*, the addition of cetuximab to FOLFIRI significantly increased the overall response rate (57.3 % vs. 39.7%, OR 2.1, 95% CI 1.5–2.9, *p* < 0.0001) and PFS (9.9 months vs. 8.4 months, HR 0.7, 95% CI 0.56–0.87, *p* = 0.0012). Median OS was also extended among patients with wild-type *KRAS* – from 20.0 months to 23.5 months (HR 0.796, 95% CI 0.67–0.95, *p* = 0.0094)^41^. Patients with *KRAS* mutations did not benefit from the addition of cetuximab.

When analyses of patients in the OPUS trial were repeated in order to evaluate the influence of *KRAS* mutation status on response to treatment^76^, only those with wild-type *KRAS* tumors benefited from the addition of cetuximab to FOLFOX. The addition of cetuximab to FOLFOX in the first-line setting improved the response rate (57 % vs. 34%, 95% CI 1.34–4.72, *p* = 0.0027), PFS (8.3 months vs. 7.2 months, HR 0.57, 95% CI 0.38–0.89, *p* = 0.0064) and OS (22.8 months vs. 18.5 months, HR 0.86 95% CI 0.60–1.22, *p* = 0.3854) compared with FOLFOX alone among mCRC patients with wild-type *KRAS* tumors. Patients with *KRAS* mutations did not benefit from the addition of cetuximab to FOLFOX and, in fact, experienced significantly reduced PFS (5.5 months vs. 8.6 months, HR 1.72, *p* = 0.02) and OS (13.4 months vs. 17.5 months, HR 1.29, *p* = 0.2) compared with those who were treated with FOLFOX alone^41^.

In the CAIRO2 trial, which showed a negative effect of cetuximab on PFS when added to a treatment regimen of capecitabine, oxaliplatin, and bevacizumab for previously untreated mCRC, among the subgroup of patients with wild-type *KRAS* tumors, PFS was not significantly different between the two groups, and there was a trend toward improved risk reduction with the addition of cetuximab. OS was not affected by the addition of cetuximab^54^.

Cetuximab has been suggested to exert antitumor effects through ADCC, which occurs as a result of interaction of the Fc portion of the antibody with Fc receptors (FcgammaRs) expressed by immune cells. Bibeau et al. explored the association of FcgammaR polymorphisms and *KRAS* mutation with clinical outcomes of patients with irinotecan-refractory mCRC treated with cetuximab plus irinotecan^77^. Tumor and normal tissues from 69 patients were screened for *KRAS* mutations and genotyped for FcgammaRIIa and FcgammaRIIIa polymorphisms. Patients with *KRAS* mutations had lower response rates (4% vs. 27%, *p* = 0.021) and shorter PFS (3.0 vs. 5.3 months, *p* = 0.021) than those with wild-type *KRAS* tumors. FcgammaRIIa-131H/H and FcgammaIIIa-158V/V genotypes were associated with longer PFS than 131R and 158F genotypes (5.5 vs. 3.0 months, *p* = 0.005) – regardless of *KRAS* status. The clinical relevance of the FcgammaR polymorphisms in this study, regardless of *KRAS* status, suggests that ADCC may be an important contributor in the mechanism of action of cetuximab.

#### 6.1.2 Panitumumab

Patients with mCRC with *KRAS* mutations are less likely to respond to therapy with panitumumab. In the phase III, randomized trial by Van Cutsem et al. comparing panitumumab and BSC in chemotherapy-refractory mCRC^57^, biomarker analyses were conducted to determine whether the effect of panitumumab monotherapy on PFS differed among patients whose tumors contained mutant *KRAS* and those whose tumors contained wild-type *KRAS*^78^. Of the 463 patients enrolled in the original study, 427 were included in the *KRAS* analysis. *KRAS* mutations were detected in 43% of patients in the panitumumab group and 40% of patients in the group receiving BSC. Patients with wild-type *KRAS* experienced a significantly greater relative improvement in PFS with panitumumab treatment versus BSC (HR 0.45; 95% CI 0.34 to 0.59). No benefit of panitumumab was seen among patients with mutant *KRAS* tumors (HR 0.99; 95% CI 0.73 to 1.36). Because of the crossover design of the study, whereby 77% of patients with mutant *KRAS* and 76% of patients with wild-type *KRAS* in the BSC group received panitumumab, an effect on OS could not be determined.

Freeman et al. determined the *KRAS* status of patients from three phase II studies of panitumumab monotherapy in the treatment of mCRC^79^. Of the 62 tumor samples available for genomic DNA sequencing, 38.7% had a *KRAS* mutation and 61.4% had wild-type *KRAS*. Wild-type *KRAS* was significantly associated with response to panitumumab (*p* = 0.0028).

The STEPP^80^ and PRECEPT^81^ studies are evaluating second-line treatment with panitumumab plus irinotecan in patients with unresectable mCRC who failed first-line treatment with oxaliplatin-based chemotherapy including bevacizumab. Both studies assessed patients for *KRAS* tumor status. STEPP randomly assigned patients to treatment with either panitumumab plus FOLFIRI or to panitumumab plus irinotecan. In the single-arm PRECEPT trial, patients received panitumumab 6 mg/kg plus FOLFIRI every two weeks until disease progression. Interim analysis have showed improved efficacy among patients with wild-type *KRAS*.

The recently completed PRIME study was the first global, phase III trial to investigate the combination of an anti-EGFR monoclonal antibody with FOLFOX as first-line treatment for patients with mCRC^60^. In PRIME, 1183 patients were randomly assigned in 1:1 ratio to biweekly treatment with either panitumumab 6.0 mg/kg plus FOLFOX4 or FOLFOX4 alone, with a primary endpoint of PFS. Secondary endpoints included OS, objective response rate, time to progression, duration of response, and safety. Patients were also stratified in each arm according to their *KRAS* mutational status to identify its applicability as a potential biomarker for panitumumab activity. When administered in combination with FOLFOX, panitumumab significantly prolonged progression-free survival (PFS) compared with FOLFOX alone (9.6 months vs. 8.0 months; HR = 0.80; 95% CI 0.66–0.97; *p* = 0.0234) in the first-line treatment of patients with *KRAS* wild-type mCRC^82^.

### 6.2 Other genetic markers of response

#### 6.2.1 BRAF mutations

As *KRAS* mutations account for only 30–40% of patients who do not respond to EGFR inhibitors^70^,^83^–^86^, additional genetic determinates of resistance to EGFR inhibitors in mCRC are needed. In the absence of *KRAS* mutations, resistance to anti-EGFR therapies is thought to be caused by alternations of other members of the RAS-RAF-MAPK pathway^87^.

Using retrospective genetic analyses of 113 tumors from cetuximab- and panitumumab-treated mCRC patients, Di Nicolantonio et al. assessed whether *BRAF* mutations affect response to treatment with these anti-EGFR MoAbs^87^. *KRAS* mutations were present in 30% of patients and, as expected, were associated with resistance to anti-EGFR therapy (*p* = 0.011). *BRAF* mutations were detected in 14% of patients with wild-type *KRAS* tumors, and were associated with nonresponse to anti-EGFR therapy (*p* = 0.029). Patients with *BRAF* mutations also had significantly shorter PFS (*p* = 0.011) and OS (*p* < 0.0001) compared with patients with wild-type *BRAF* tumors.

Recently, Ruzzo et al. retrospectively assessed *KRAS* and *BRAF* mutational status in 117 irinotecan-refractory EGFR-positive mCRC patients treated with cetuximab plus irinotecan^88^. Among the 66 patients with wild-type *KRAS*, nine had *BRAF* mutations and 57 had wild-type *BRAF* tumors. Those with wild-type *BRAF* experienced significantly improved RR (0% vs. 33%, *p* = 0.04) and PFS (3.3 vs. 5.1 months, *p* = 0.076; HR=0.54; 95% CI 0.18–1.09) compared with those with mutated *BRAF*.

In a recent report of 276 chemorefractory mCRC patients treated with cetuximab (with or without irinotecan), those with a combined wild-type *KRAS*, *BRAF* and *NRAS* status experienced a significantly greater response, progression-free survival, and OS compared with patients with a mutation in one of these genes^89^. Mutations of *KRAS*, *BRAF* and *NRAS* were shown to be mutually exclusive and present in at least 47% of mCRC patients^89^.

In a recent analysis of the CAIRO2 trial, which evaluated the efficacy of chemotherapy plus bevacizumab with or without the addition of cetuximab in patients, patients with *BRAF* mutated tumors were found to have decreased median PFS and OS compared with patients with wild-type tumors, irrespective of the treatment arm^90^. These results suggest that, in contrast to *KRAS* mutations, the association of *BRAF* with outcome is not restricted to patients treated with cetuximab.

#### 6.2.2 Overexpression of epiregulin and amphiregulin

Overexpression of the EGFR ligands epiregulin and amphiregulin in primary tumors has been associated with favorable outcomes in mCRC patients treated with cetuximab^71^,^91^ – a finding that is limited to patients with wild-type *KRAS* tumors^91^,^92^. In the NCIC CTG CO.17 study, tumor samples were analyzed and the cohort was subdivided into those with low or high EREG. Results demonstrated that OS was better for cetuximab than BSC among patients with high EREG (HR 0.43; *p* < 0.0001) but not for low EREG patients (HR 0.77, *p* = 0.28)^93^. High EREG AND KRAS WT status (“Combimarker”) was present in 139 (36%). Within the Combimarker-positive group, the median PFS was 5.4 vs. 1.9 months (HR, 0.31; *p* < 0.0001), and median OS 9.8 vs. 5.1 months (HR, 0.43; *p* < 0.001) in the cetuximab vs. BSC arms, respectively. In the rest (n = 246, 64%), cetuximab was not associated with improved PFS (HR, 0.82; *p* = 0.12) or OS (HR, 0.90; *p* = 0.45)^93^.

It is likely that tumors overexpressing these markers are more dependant on EGFR activation and therefore more likely to respond to anti-EGFR MoAbs^94^.

#### 6.2.3 EGFR gene copy number

EGFR gene copy number has also shown to be correlated with response to anti-EGFR MoAb therapy^95^,^96^. In a report of 31 patients with mCRC treated with cetuximab or panitumumab, those who responded to anti-EGFR therapy had a median EGFR gene copy number of 6.8 per cell compared with a median of 1.93 in nonresponders^83^.

In the phase III trial comparing panitumumab plus BSC and BSC alone, a subset of patients had tumor samples adequate to determine gene copy number by FISH. Among the 58 patients treated with panitumumab for mCRC, 2.47 was identified as the cutoff gene copy number for response, with an overall accuracy of 75.9% (95% CI, 62.8% to 86.1%)^95^. No patient with an EGFR gene copy number below 2.47 had a tumor response, while six of 20 patients with an EGFR gene copy number above 2.47 had an objective response (*p* = 0.0009).

In a retrospective analysis of 85 chemorefractory mCRC patients, a gene copy number of 2.92 was found to be predictive of response to treatment with cetuximab-based therapy^96^. A response rate of 32.6% was observed among those with a gene copy number higher than 2.92, compared with a response rate of 2.4% in those with a gene copy number less than 2.92.

The combination of gene copy number and *KRAS* status may be more predictive of response to anti-EGFR therapy than *KRAS* alone^97^. In a study of 87 patients with mCRC treated with cetuximab, either alone or in combination with irinotecan, patients with wild-type *KRAS* tumors and a gene copy number higher than 2.83 were less likely to progress than those with a lower gene copy number^97^. An unexpected result was that gene copy numbers higher than 2.83 were associated with a higher likelihood of progression among patients with mutant *KRAS* tumors. These findings require validation in a large prospective trial.

#### 6.2.4 PTEN

PTEN is a key tumor suppressor that inactivates PI3K, a downstream effector of the EGFR cascade. Mutations resulting in PTEN loss lead to uncontrolled activation of PI3K/AKT signaling pathway, which may result in resistance to EGFR-blockade. The role of PTEN immunoreactivity (IHC) loss and of pAKT IHC in predicting the activity of treatment with a combination of cetuximab plus irinotecan was investigated in the primary tumors and metastases of 102 EGFR-positive mCRC patients who had undergone previous treatment with irinotecan^98^. Loss of PTEN IHC in metastases, but not primary tumors, was predictive of response to treatment and was associated with greater PFS. pAKT IHC was not associated with response in primary tumors nor in metastases.

#### 6.2.5 EGF/EGFR polymorphisms

EGF and EGFR polymorphisms are also potential markers of response to anti-EGFR therapy. Among 110 patients treated with cetuximab and irinotecan following disease progression with first-line FOLFOX and a second-line irinotecan-based regimen, patients with a short-repeat variant of the highly polymorphic CA dinucleotide repeat in intron-1 of the EGFR gene were more responsive to treatment and had an increased rate of skin rash than patients with a long-repeat variant of CA^99^. In experimental models, decreasing the number of CA pairs has been shown to enhance EGFR transcription^100^; this may render tumors more dependent on the EGF pathway and, therefore, more responsive to EGFR inhibition.

Garm-Spindler et al. investigated the methodological aspects of *KRAS* testing, and the predictive and prognostic value of *KRAS* status combined with three EGFR-related gene polymorphisms in mCRC patients treated with third-line cetuximab and irinotecan^101^. Response was limited to patients with wildtype *KRAS* tumors, translating to a significantly higher PFS. The *EGF*61A>G polymorphism was found to be significantly associated with clinical outcome in patients with wild-type *KRAS* mutations. Patients with homozygous *EGF*61 tumors had a significantly lower rate of progression (19% vs. 60%, *p* = 0.006) and a significant increase in OS (17.1 vs. 5.9 months, *p* = 0.002) in comparison with those with *EGF*61A/G tumors. Combined biomarker analysis to determine *KRAS* and *EGF*61 status may therefore offer an additional approach to the selection of patients for third-line treatment involving anti-EGFR therapies.

### 6.3 Papulopustular rash

EGFR blockade by cetuximab or panitumumab alters the mediation of epidermal basal keratinocytes by EGFR, resulting in the characteristic papulopustular rash. In most clinical trials, the incidence and severity of the rash has been well correlated with a clinical benefit of anti-EGFR therapy^33^,^55^,^57^,^58^.

In the BOND trial, clinical response rates were higher among patients who experienced skin reactions in response to cetuximab treatment (25.8% vs. 6.3% in the combination therapy group [*p* = 0.005] and 13.0% vs. 0% in the monotherapy group)^20^.

**Figure 3:**
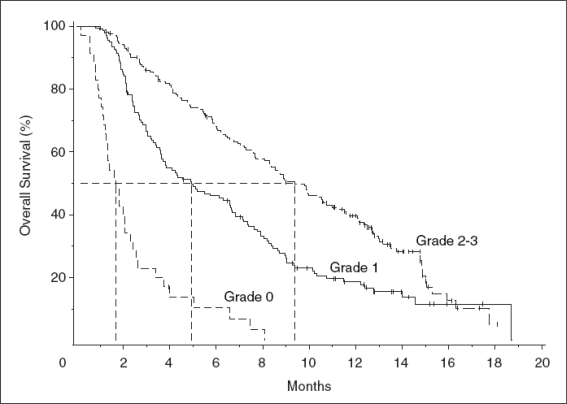
Association between progression-free survival and severity of rash in patients with colorectal cancer treated with cetuximab^32^

Among 346 patients with EGFR-expressing mCRC refractory to irinotecan, oxaliplatin, and fluoropyrimidines, OS among patients treated with cetuximab correlated strongly with the presence and severity of rash^32^. Grade 1 and grade 2–3 rash were associated with median survival times of 4.9 months (95% CI 3.6–6.6 months) and 9.4 months (95% CI 8.1–11.0), respectively (Fig. 3), compared with 1.7 months (95% CI, 1.2 to 2.3) for patients without rash. Response was also strongly correlated with the presence and severity of rash. Of the 311 patients who developed rash, 13% (95% CI 9.3–17.1%) experienced a partial response. Partial responses were observed in 7%, 17%, and 20% of patients with grade 1, 2, or 3 rashes, respectively, and in none of the 35 patients without rash.

The presence and severity of rash has also been correlated with clinical efficacy in trials of panitumumab. In the phase III trial comparing panitumumab and BSC in the treatment of mCRC, PFS appeared to favor patients with grade 2–4 skin toxicity versus those with grade 1 skin toxicity (HR 0.62; 95% CI 0.44–0.88)^57^. There was also a trend toward longer OS in patients with grade 2–4 skin toxicity compared with patients with grade 1 skin toxicity (HR 0.70, 95% CI 0.47–1.05)^58^.

Data from the recent EVEREST trial (Evaluation of Various Erbitux Regimens by Means of Skin and Tumor Biopsies), involving patients with no or limited skin reactions, suggest that individualized dose titration based on the occurrence and severity of rash might be used to improve treatment response to EGFR inhibitors^102^. However, as the rash only appears after treatment, its value as a predictive marker is somewhat limited^94^.

## 7. CONCLUSIONS

The anti-EGFR therapies cetuximab and panitumumab have both demonstrated clinical efficacy in monotherapy in patients with mCRC, an advantage that has recently been found to be limited largely to those with wild-type *KRAS* tumors. The advantages of using these agents in monotherapy include reduced cost and toxicity. The addition of cetuximab to irinotecan results in superior PFS and RR compared with cetuximab monotherapy, and is the preferred regimen for patients with irinotecan-resistant mCRC. There is currently no evidence for a benefit of panitumumab in combination with irinotecan.

In the first-line setting, cetuximab has been shown to improve PFS and response rate in patients with wild-type *KRAS* tumors, in combination with FOLFOX and FOLFIRI chemotherapy. Cetuximab has also been shown to improve the potential for curative resections when added to chemotherapy (FOLFOX or FOLFIRI). Used alone in the third-line setting, cetuximab has also been shown to improve OS and PFS in patients with wild-type *KRAS* tumors^33^,^73^.

Despite the efficacy of the anti-EGFR MoAbs in the treatment of mCRC, response rates remain suboptimal. In order to optimize treatment with these targeted therapies, it is important to select the patients who are most likely to benefit from treatment. Several biologic markers involved in EGFR intracellular signaling pathways have been investigated as potential predictors of response to the anti-EGFR MoAbs. The most relevant of these markers is *KRAS*, and patients with *KRAS* mutations do not benefit from treatment with cetuximab or panitumumab. Other markers, such as *BRAF*, epiregulin and amphiregulin, EGFR gene copy number, PTEN, and EGF/EGFR polymorphisms, are currently under investigation and show promise but are considered experimental at this time^94^.

While cetuximab is currently administered according to a weekly dosing schedule, pharmacokinetic data indicate that it has a long terminal half-life, potentially allowing administration on a biweekly basis. A biweekly dosing schedule would allow greater treatment flexibility when combined with biweekly or longer chemotherapy regimens, as well as potential cost savings. Results from phase I studies show that a biweekly schedule of cetuximab is well tolerated, and exhibits similar pharmacokinetics and pharmacodynamics to conventional weekly dosing, without compromising efficacy^64^,^103^. Panitumumab, which is currently approved for biweekly administration, has been evaluated for administration every three weeks. Although pharmacokinetic data suggest that it may be flexibly dosed from weekly to every three weeks^68^, other data suggest higher rates of several grade 3 adverse events (erythema, pruritus, acneiform dermatitis, fatigue, hypomagnesemia) with this regimen^69^. Further evaluation of alternative dosing regimens for cetuximab and panitumumab is needed due to the small number of patients in these studies.

## Figures and Tables

**Table I.** Efficacy of treatment with weekly cetuximab and irinotecan (CetIri) or biweekly CetIri in two consecutive periods^64^

*Characteristic*	*Weekly CetIri 250 mg/m^2^*	*Biweekly CetIri 500 mg/m^2^*
Number	65	74
Time from nonresectable disease to ‘indication for cetuximab’, months (range)	20 (5–58)	16 (3–60)
Median ‘delay’ time, time from date of indication to the first infusion of CetIri, weeks (range)	6 (0–88)	6 (0–36)
Median number of weeks with cetuximab (range)	16 (1–51)	17 (2–52) (8 biweekly courses)
Response rate		
Complete response (CR)	0	1 (1%)
Partial response (PR)	12 (19%)	18 (24%)
Stable disease (SD)	31 (47%)	38 (52%)
Disease control (CR + PR + NC)	43 (66%)	57 (77%)
Progression (PD)	15 (23%)	13 (18%)
Not evaluable	7 (11%)	4 (5%)
PFS, months (95% CI)	5.4 (4.6–6.1)	5.4 (4.7–6.5)
OS, months (95% CI)	10.4 (7.2–13.1)	8.9 (7.0–10.5)

NC = No change; PFS = Progression-free survival; OS = Overall survival
